# Hearing Care Disparities in Dementia: Access and Usability in the Coming Era of OTC Hearing Aids

**DOI:** 10.1093/geroni/igaa057.2686

**Published:** 2020-12-16

**Authors:** Carrie Nieman, Jennifer Deal, Sara Czaja, Esther Oh

**Affiliations:** 1 Johns Hopkins University School of Medicine, Baltimore, Maryland, United States; 2 Johns Hopkins Bloomberg School of Public Health, Baltimore, Maryland, United States; 3 Weill Cornell Medicine, New York, New York, United States

## Abstract

Age-related hearing loss is highly prevalent among persons with dementia (PwDs) and is associated with an increased risk of neuropsychiatric symptoms. However, few use hearing aids and disparities exist. PwDs and, in particular, minority older adults, have some of the lowest rates of hearing aid use. Recent federal legislation created the designation of over-the-counter hearing aids, which will debut by 2020-2021, and represents an opportunity to advance accessibility. This presentation will share estimates of hearing aid use among community-dwelling PwDs from two cohorts, where hearing aid use ranges from 7-11% among African Americans versus 33-45% among whites. To explore this gap, the presentation will share findings from semi-structured interviews with care partners of PwDs and hearing loss around barriers and facilitators of hearing care, including device usability. With growing understanding of sensory health, a changing hearing care landscape represents a critical opening to increase access to hearing care for PwDs. Part of a symposium sponsored by the Alzheimer’s Disease Research Interest Group.

